# 

**Published:** 1882-11

**Authors:** 


CONTENTS:

ART. I.—Specialties and their Advantages. By Geo. A. Mills, D. D S. ............ 289
ART. II.—Histology of the Nerves (Continued) ............ 308
ART. III.—Answers to Questions on the Teeth of the White, Mixed and Black Races.. 322
ART. IV.—Extraction of Teeth in Pregnant Women ............ 327
American Academy of Dental Science ............ 332

EDITORIAL, ETC.

Dental Education ............ 333

MONTHLY SUMMARY.

Zinc vs. Babbitt Metal ............ 334
Acute Conjunctivitis Caused by the Electric Light ............ 335
Nitrous Oxide ............ 336

